# Supplementary material for: Implication for alphavirus host-cell entry and assembly indicated by a 3.5Å resolution cryo-EM structure
Source: Nat Commun. 2018 Dec 14;9:5326. doi: 10.1038/s41467-018-07704-x (PMC6294011; doi:10.1038/s41467-018-07704-x)
Supplement: Supplementary file 1 — Supplementary Information [file 41467_2018_7704_MOESM1_ESM.pdf]

**Supplementary Materials:**

Supplementary Figures 1-4

Supplementary Tables 1-3

# Implication for alphavirus host-cell entry and assembly indicated by a 3.5 Å resolution cryo-EM structure

**Authors:** Lihong Chen[1,5†], Ming Wang[2†], Dongjie Zhu[1,3†], Zhenzhao Sun[2†]， Jun Ma[1], Jinglin Wang[4], Lingfei Kong[1], Shida Wang[2], Zaisi Liu[2], Lili Wei[2], Yuwen He[4], Jingfei Wang[2]* and Xinzheng Zhang[1,5,6]*

[1]National Laboratory of Biomacromolecules, CAS Center for Excellence in Biomacromolecules, Institute of Biophysics, Chinese Academy of Sciences, Beijing 100101, P.R. China.

[2]State Key Laboratory of Veterinary Biotechnology, Harbin Veterinary Research Institute, Chinese Academy of Agricultural Sciences, Harbin 150069, P.R. China.

[3]School of Life Science, University of Science and Technology of China, Hefei 230026, P.R. China.

[4]Yunnan Tropical and Subtropical Animal Viral Disease Laboratory, Yunnan Animal Science and Veterinary Institute, Kunming 650224, P.R. China.

[5]University of Chinese Academy of Sciences, Beijing 100049, P.R. China.

[6]Lead author : Xinzheng Zhang

[†]These authors contributed equally to this work.

*Correspondence to: wangjingfei@caas.cn (J.F.); xzzhang@ibp.ac.cn (X.Z.)

# Supplementary Materials

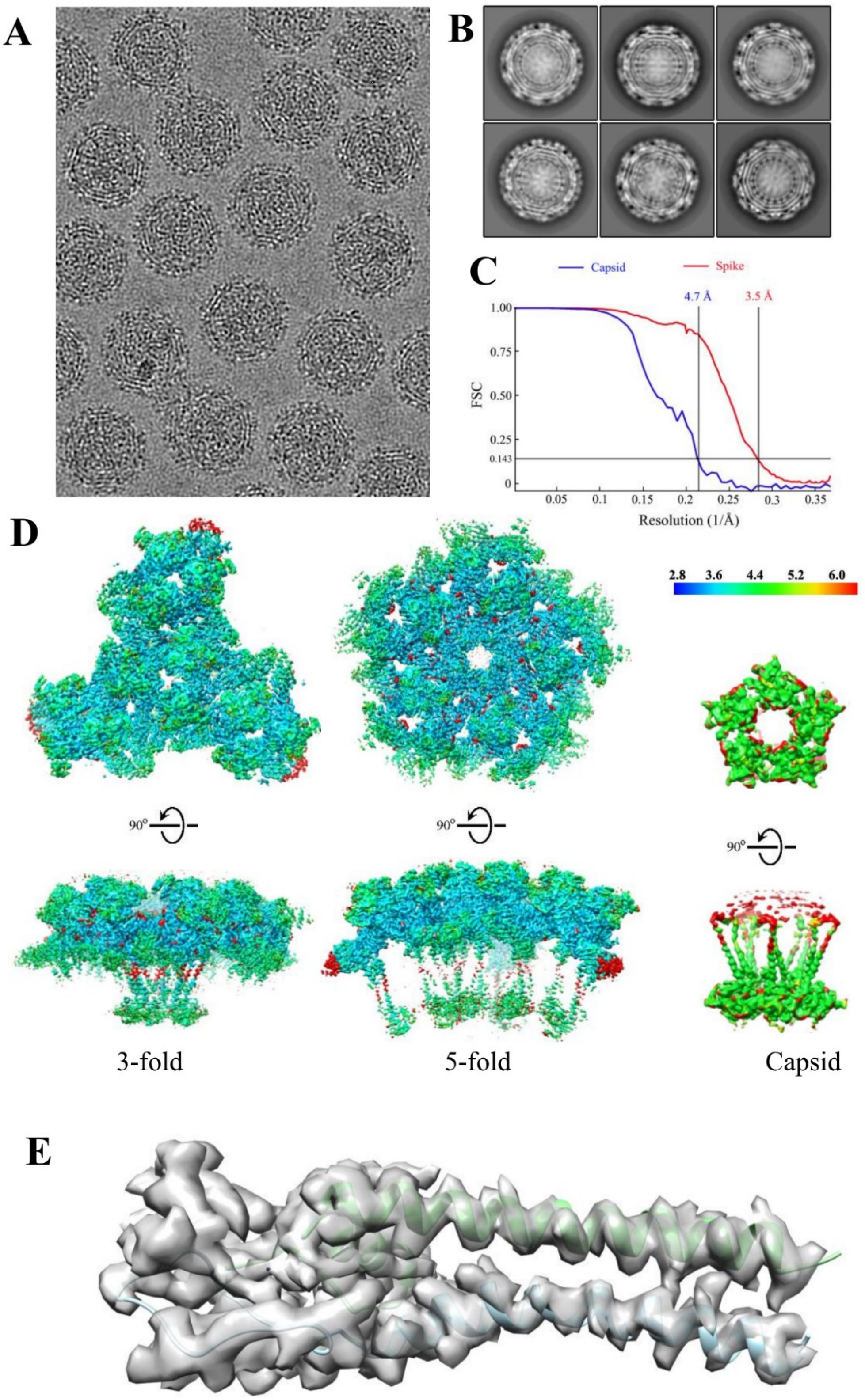

**Supplementary Figure 1.** cryo-EM reconstruction of SINV. (A) A representative micrograph of SINV. (B) Reference free 2D class averages. (C) Fourier Shell Correlation Coefficient (FSC) of glycoprotein shell (red) and NC (blue) is shown. (D) Local resolutions of glycoprotein shell and NC. (E) The cryo-EM densities of the hydrophobic pocket and the TMs.

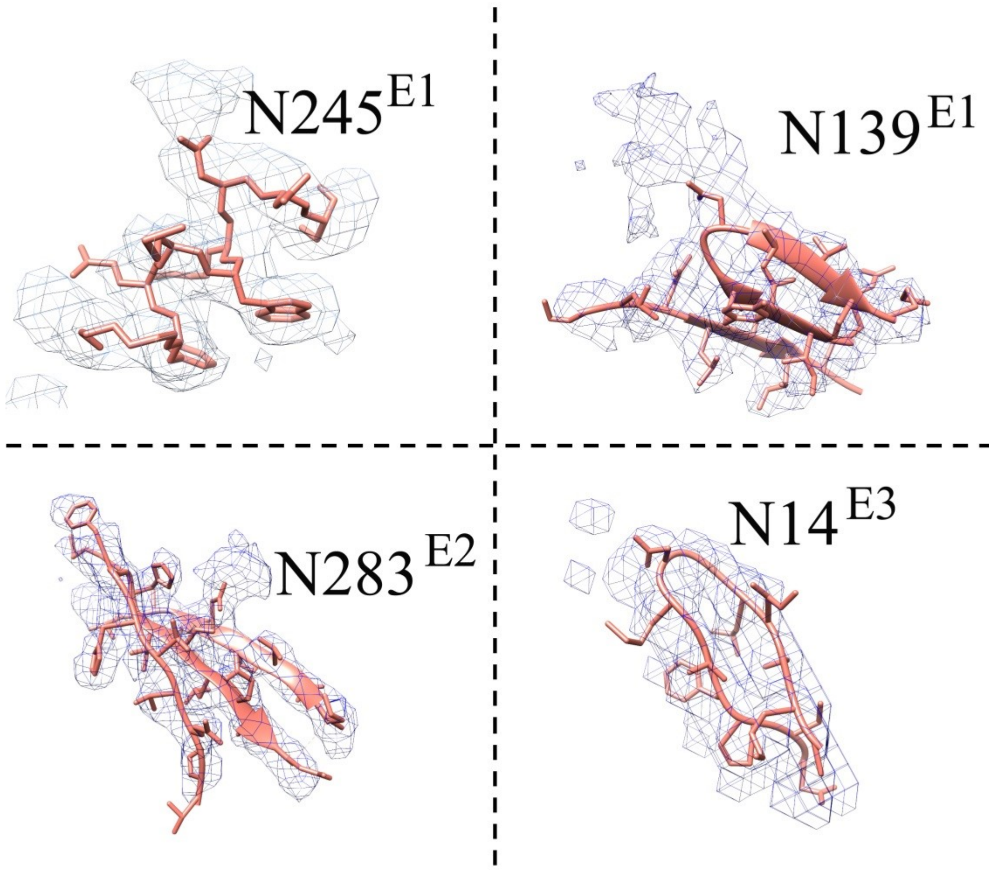


**Supplementary Figure 2. Glycosylation sites in the SINV.**

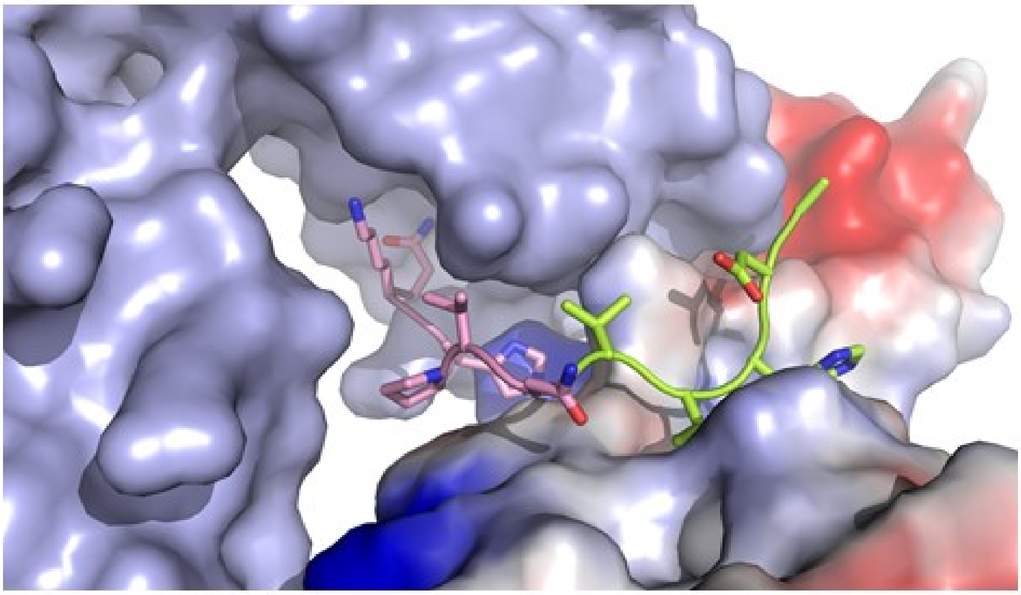

**Supplementary Figure 3. Stem loop.** The stem loop (depicted in ribbon) from P383 to V388 is associated with E2 domain (green), while N389 to N394 (colored by pink) bridges E1 and E2 enhancing the interactions between E1 and E2. E1 is shown in purple and E2 is colored by surface potential.

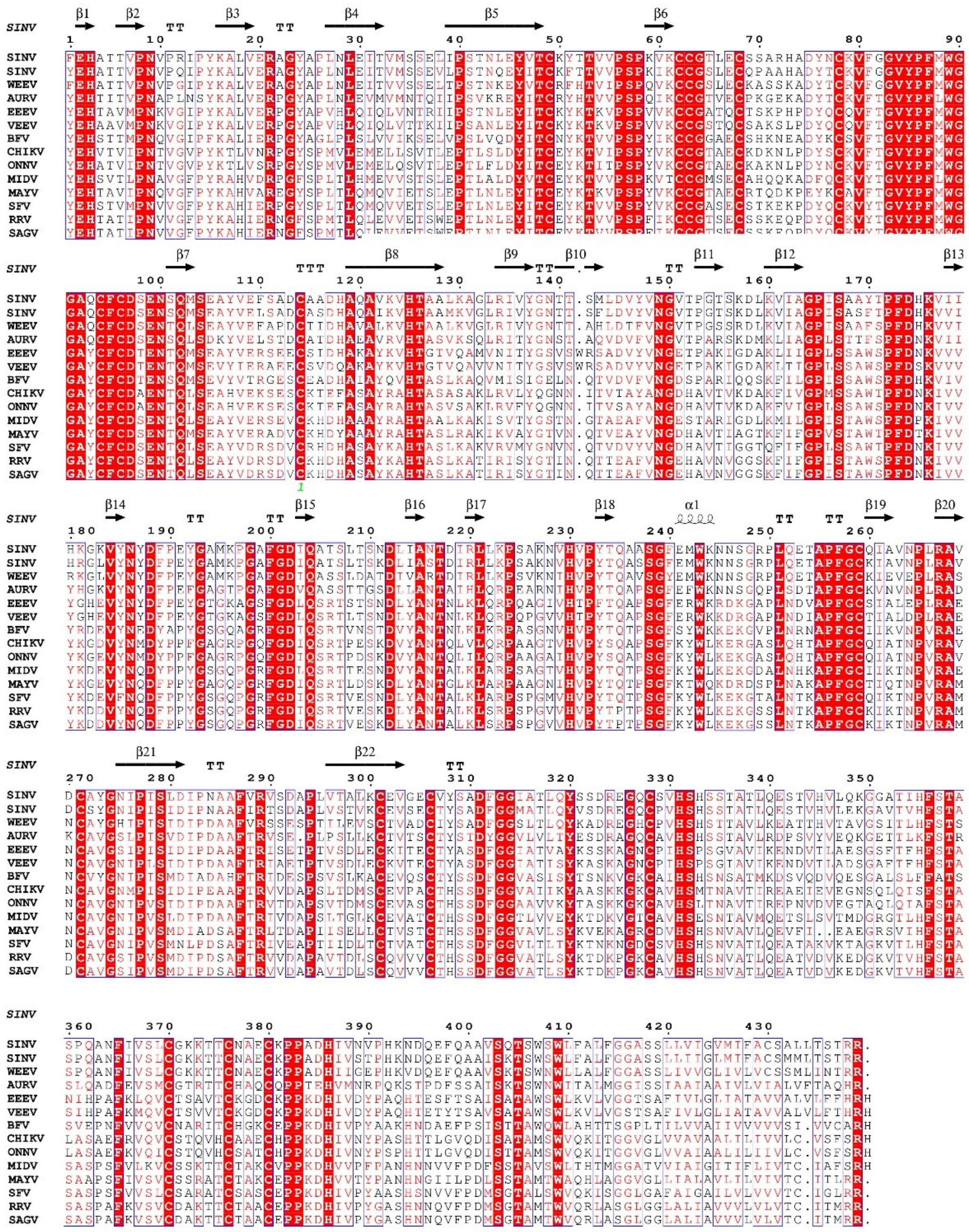

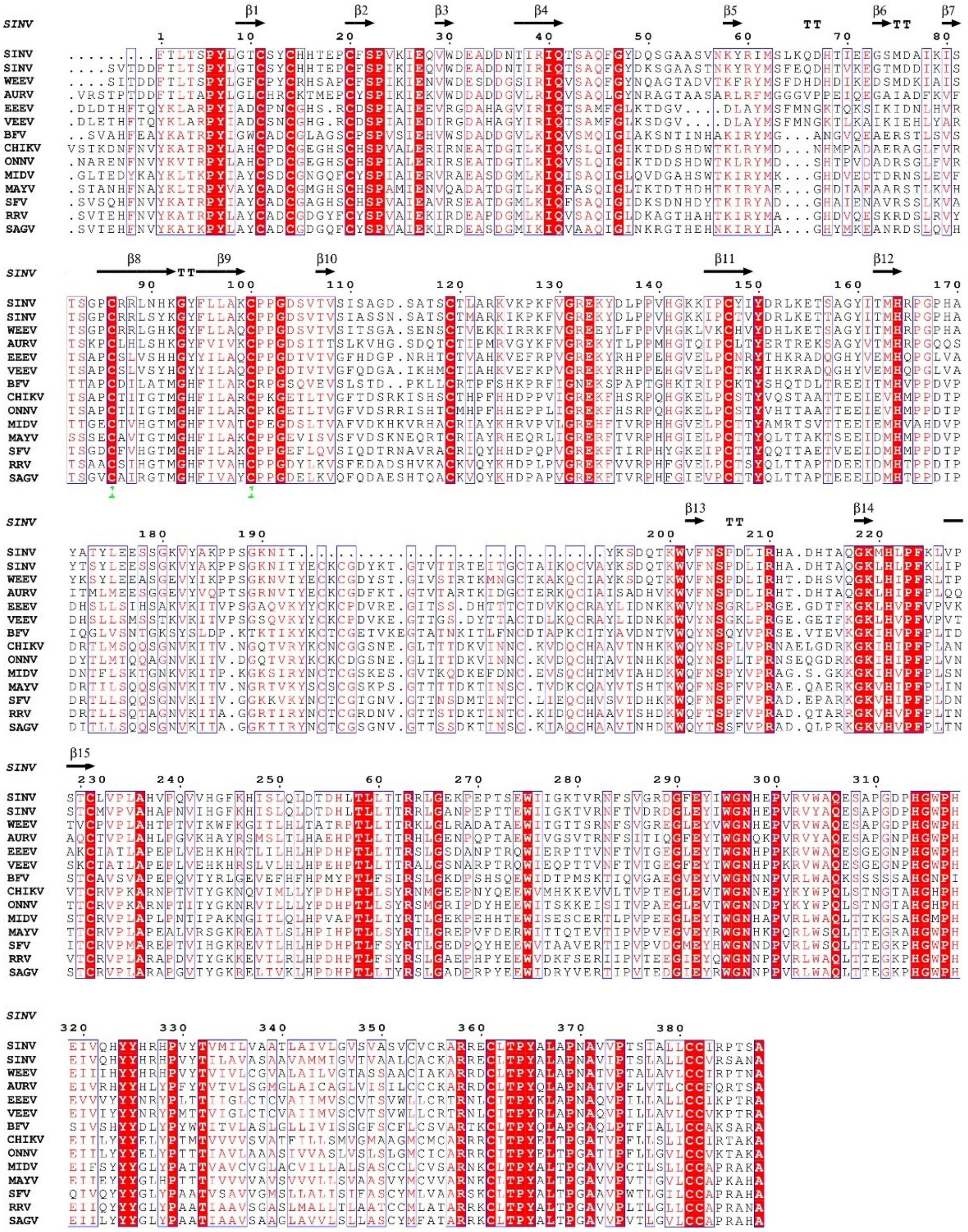
SINV
β1 β2 β3 β4 β5 β6 β7 β8 β9 β10 β11 β12 β13 β14 β15
TT
SINV
SINV
WEEV
AURV
EEEV
VEEV
BFV
CHIKV
ONNV
MIDV
MAYV
SFV
RRV
SAGV

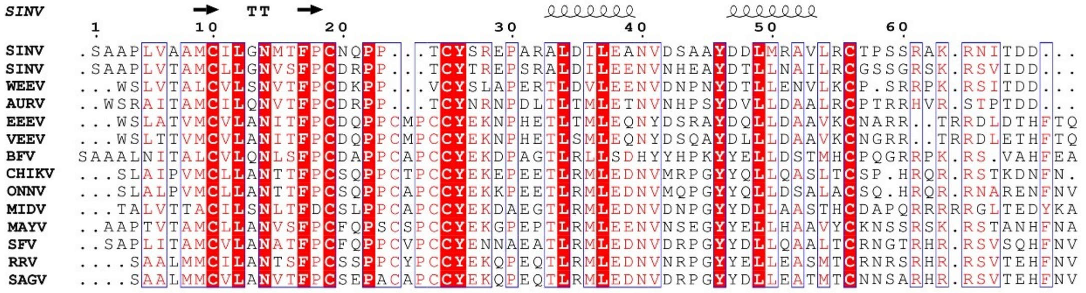


**Supplementary Figure 4. Sequence alignment of E1, E2 and E3 from representative members of alphavirus, respectively.** The secondary structure elements are displayed above the sequences. Fully conserved residues and similar residues are shaded and shown in red, respectively. Sequences are from SINV (Sindbis virus, this study), SINV (Sindbis virus, AAA96976.1), WEEV (Western equine encephalitis virus, AAA42999.1), AURV (Aura virus, AAD13623.1), EEEV (Eastern equine encephalitis virus, AAT96380.1), VEEV (Venezuelan equine encephalitis virus, ABB45866.1), BFV (Barmah Forest virus), CHIKV (Chikungunya virus, CAJ90470.1), ONNV (O'nyong nyong virus, AAC97205.1), MIDV (Middleburg virus, AA033343.1), MAYV (Mayaro virus, AAO33335.1), SFV (Semliki Forest virus, CAA27742.1), RRV (Ross River virus, AAA47404.1), and SAGV (Sagiyama virus, AAO33337.1).

**Supplementary Table 1. Comparison of E1/E2 heterodimer with SINV, CHIKV crystal structures and VEEV cryo-EM structure of E1/E2 heterodimer**

| **E1** **RMSD/Å** | SINV This study | SINV (3MUU) | CHIKV (3N44) |
|---|---|---|---|
| SINV(3MUU) | 1.918 | | |
| CHIKV (3N44) | 2.053 | 2.639 | |
| VEEV (3J0C) | 1.491 | 2.206 | 1.839 |
| **E2** **RMSD/Å** | SINV This study | SINV (3MUU) | CHIKV (3N44) |
| SINV(3MUU) | 1.385 | | |
| CHIKV (3N44) | 2.304 | 1.567 | |
| VEEV (3J0C) | 1.836 | 1.461 | 1.820 |

**Supplementary Table 2. The E1 amino acids that form contacts in type I and type II inter-triangle interactions.** The completely conserved amino acids are highlighted in footnote 1 and the highly conserved amino acids are highlighted in footnote 2.

| Type of interaction | Contact region | quasi-three-fold | three-fold |
|---|---|---|---|
| **Type I** | Region III | **H125[1]——S41[2]**<br>**S41[2]——H125[1]** | |
| | Region II/IV | **Y192[2]——Y147[2]**<br>**A194[2]——T152[2]** | |
| | Region I/V | **A22[2]——V307[2]**<br>**V290[2]——I315[2]** | |
| **Type II** | Region I | **L295[1]——I315[1]**<br>**L295[1]——T317[1]** | |

**Supplementary Table 3. Cryo-EM data collection, refinement and validation statistics**

| | Envelope glycoprotein EMDB-9693 PDB 6IMM | Capsid core EMDB-9692 |
|---|---|---|
| **Data collection and processing** | | |
| Magnification | 18000 | |
| Voltage (kV) | 300 | |
| Electron exposure (e–/Å$^2$) | 50 | |
| Defocus range (μm) | 1.8-2.5 | |
| Pixel size (Å) | 0.68 | |
| Symmetry imposed | icosahedron | |
| Initial particle images (no.) | 36318 | |
| Final particle images (no.) | 29974 | |
| FSC threshold | 0.143 | |
| Map resolution range (Å) | 2.8-6.4 | |
| Map resolution (Å) | 3.5 | |
| **Refinement** | | |
| Initial model used (PDB code) | 3MUU/3N40 | |
| Model resolution (Å) | 3.29/2.17 | |
| FSC threshold | | |
| Map sharpening *B* factor (Å$^2$) | -170 | |
| R.m.s. deviations | | |
| Bond lengths (Å) | 0.012 | |
| Bond angles (°) | 1.50 | |
| Validation | | |
| MolProbity score | 6.13 | |
| Clashscore | 6.37 | |
| Poor rotamers (%) | 1.80 | |
| Ramachandran plot | | |
| Favored (%) | 85.05 | |
| Allowed (%) | 14.6 | |
| Disallowed (%) | 0.35 | |
